# Temporal expression dynamics of glypicans during hiPSC cardiac differentiation

**DOI:** 10.3389/fcell.2026.1778977

**Published:** 2026-02-25

**Authors:** Fernanda C. P. Mesquita, Stephanie J. Kim, Andreia Z. Chignalia, Camila Hochman-Mendez

**Affiliations:** 1 Regenerative Medicine Research, The Texas Heart Institute at Baylor College of Medicine, Houston, TX, United States; 2 Michael E. DeBakey Department of Surgery, Baylor College of Medicine, Houston, TX, United States; 3 McGovern Medical School, The University of Texas Health Science Center at Houston, Houston, TX, United States; 4 Department of Internal Medicine, Division of Pulmonary, Critical Care and Sleep Medicine, College of Medicine, University of Arizona, Phoenix, AZ, United States; 5 Biofluids Repository Core, Advanced Technology Core, Baylor College of Medicine, Houston, TX, United States

**Keywords:** cardiac differentiation, dynamic expression, gene expression, glypicans, human pluripotent stem cells

## Abstract

Human pluripotent stem cells (hPSCs) offer a versatile platform for modeling human cardiac development and generating cardiomyocytes for research and translational applications. Cardiac differentiation protocols are well established and rely on the sequential activation and inhibition of WNT, BMP, and FGF signaling pathways to guide lineage progression. While these intracellular signaling events are well characterized, less attention has been given to the temporal behavior of extracellular components present at the cell surface during differentiation. Glypicans (GPCs) are a family of membrane-bound heparan sulfate proteoglycans within the glycocalyx that are known to interact with morphogens in multiple developmental contexts. In this study, we profiled the expression of GPC1-6 during a standard chemically defined cardiac differentiation protocol, in the absence of targeted interventions. Gene expression analysis across stages revealed distinct, stage-associated patterns: GPC3 and GPC6 were upregulated during the WNT activation phase; GPC4 was suppressed after WNT inhibition and maintained low during cardiac commitment. GPC2 and GPC5 expressions peaked during the formation of cardiac progenitors, and GPC1 expression increased following cardiac specification. These findings provide a temporal map of GPC expression coinciding with established differentiation stages, demonstrating that members of the glypican family are dynamically expressed during human cardiac differentiation. By documenting when specific glypicans are expressed during a commonly used differentiation workflow, this study offers a descriptive reference framework that may inform future mechanistic studies investigating how extracellular components intersect with canonical cardiac signaling pathways.

## 1 Introduction

Human pluripotent stem cells (hPSCs) provide an unprecedented platform for modeling human heart development, studying disease mechanisms, and advancing regenerative medicine. Directed differentiation of hPSCs into the cardiac lineage has significantly transformed cardiovascular research by enabling the generation of patient-specific cardiomyocytes (Mummery et al., 2012). Over the past 2 decades, substantial progress has been made in refining differentiation strategies. Early embryoid body–based methods produced low (<10%) and variable cardiomyocyte yields under nonselective conditions (Zhang et al., 2009). A major advance came with the recognition that cardiogenesis follows a tightly ordered sequence of morphogenetic cues—most prominently the wingless/int (WNT) proteins, bone morphogenetic proteins (BMPs), and fibroblast growth factor (FGF) signaling pathways. By temporally and quantitatively mimicking these signals, researchers have established chemically defined, growth factor–controlled protocols that consistently produce high cardiomyocyte yields (>90%) across multiple hPSC lines (Huang et al., 2025), bringing *in vitro* cardiac differentiation closer to developmental fidelity.

Despite these advances, hPSC-derived cardiomyocytes still lack the structural, metabolic, and electrophysiological maturity of adult heart cells. This gap suggests that, beyond canonical morphogen signaling, additional regulatory mechanisms may fine-tune lineage specification and maturation. Growing evidence highlights the importance of extracellular microenvironment, particularly the composition and architecture of the extracellular matrix (ECM) and its associated molecular interfaces, in modulating morphogen pathways during cardiac differentiation (Romano, 2025). The ECM functions not only as a structural scaffold but also as a dynamic biochemical network that anchors cells and provides essential instructive cues, highlighting how extracellular architecture orchestrates intracellular signaling dynamics (Wang et al., 2025).

Between the ECM and the plasma membrane lies another, often overlooked regulatory layer: the glycocalyx. This dense and dynamic meshwork of proteoglycans, glycoproteins, and glycolipids coats the cell surface, forming a biochemical interface that translates extracellular signals into intracellular ones. Far from a passive barrier, the glycocalyx functions as a signal-processing layer, controlling the accessibility, diffusion, and spatial organization of morphogens, growth factors, and cytokines. Its components can thus modulate not only the magnitude but also the timing of pathway activation (Rana et al., 2026).

Among the key constituents of the glycocalyx, glypicans (GPCs)—a conserved family of 6 heparan sulfate (HS) proteoglycans (GPC1-6)—are particularly intriguing regulators of morphogen signaling. Anchored to the cellular membrane via glycosylphosphatidylinositol (GPI) links, GPCs present HS chains that bind and modulate key signaling molecules such as WNTs, BMPs, FGFs, and Hedgehog proteins (Filmus, 2023). Through these interactions, GPCs can either enhance or restrain signaling in a context-dependent manner, effectively acting as molecular “rheostats” that fine-tune morphogen gradients and developmental outcomes.

Although GPCs are well studied in organogenesis, tumorigenesis, and angiogenesis, their roles in hPSC differentiation remain largely unexplored (Okada et al., 2019; Ueda et al., 2020; Corti et al., 2021; Piao et al., 2025). This knowledge gap is striking because the same signaling pathways modulated by GPCs—WNT, BMP, and FGF—are essential for cardiogenesis. Evidence from animal models highlights their developmental importance: Gpc4-deficient zebrafish develop severe cardiac malformations due to dysregulated WNT and BMP signaling (Strate et al., 2015), and Gpc6-deficient mice develop congenital heart defects associated with impaired FGF and Hedgehog activity (Mead et al., 2024). These studies collectively emphasize a conserved role for GPCs in regulating cardiac morphogen activity across species. In addition to development, some studies implicate GPCs in tissue regeneration, particularly GPC1, GPC2, and GPC3 in neuronal and liver regeneration (Liu et al., 2009; Abaskharoun et al., 2010; Liu et al., 2010; Ouchida et al., 2023) GPC1 has been linked to cell-cycle control and survival through nuclear growth factor signaling and cell-cycle regulation in brain endothelial cells (Qiao et al., 2008), whereas GPC3 negatively regulates liver regeneration, as its suppression enhances hepatocyte proliferation *in vitro* and *in vivo* (Liu et al., 2009; Liu et al., 2010).

Given these observations, we hypothesize that GPCs may act as stage-specific modulators of morphogen signaling during human cardiac development, potentially contributing to the temporal regulation of lineage specification and functional maturation. To explore whether GPCs are dynamically expressed during cardiac lineage commitment, we profiled their temporal expression across key stages of hiPSC cardiac differentiation.

## 2 Methods

### 2.1 Human induced pluripotent stem cell expansion and cardiac differentiation

**FIGURE 1:**
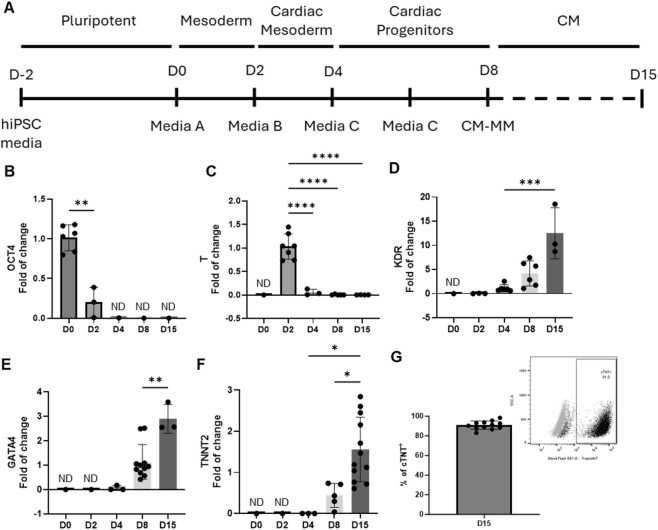
Cardiac differentiation and characterization. **(A)** Schematic diagram outlining the key steps of cardiac differentiation, including the developmental cellular stages. **(B–F)** (n = 3–12). Dynamic gene expression profiles of stage-specific markers: OCT4 (pluripotency, B), T/Brachyury (mesoderm, C), KDR (cardiac mesoderm, D), GATA4 (cardiac progenitors, E), and TNNT2 (cardiomyocytes, F). **(G)** Percentage of cTNT + cells at day 15 of differentiation (n = 12), with a representative dot plot (cTNT, black; isotype, gray). *p < 0.05, **p < 0.01, ***p < 0.001, and ****p < 0.0001. CM, cardiomyocyte; CM-MM, cardiomyocyte maturation media; D, day; ND, not detected.

The cardiomyocytes used in this study were differentiated from hiPSCs (SCVI20) with a commercially available kit (STEMCELL Technologies Inc., Cambridge, MA, United States), following established protocols (Mesquita et al., 2024) (Figure 1A). Briefly, 1.2 × 10^6^ hiPSCs were plated in 6-well tissue culture plates coated with hESC-qualified Matrigel (Corning, Bedford, MA, United States) and maintained in Essential 8™ Medium (Gibco, Grand Island, NY, United States) supplemented with 10 µM of Y-27632 (STEMCELL Technologies Inc.). The next day, the medium was replaced with fresh Essential 8™ Medium.

Cardiac differentiation was initiated on day 0 by switching to STEMdiff Cardiomyocyte Differentiation Medium A to induce mesoderm formation. On day 2, the medium was replaced with STEMdiff Cardiomyocyte Differentiation Medium B to promote cardiac mesoderm specification, followed by STEMdiff Cardiomyocyte Differentiation Medium C on days 4 and 6 to drive cardiac lineage commitment. From days 8–10, cells were maintained in STEMdiff Cardiomyocyte Maintenance Medium. On day 10, cardiomyocytes were metabolically selected for 5 additional days in glucose-free RPMI 1640 Medium (Gibco) supplemented with 1% sodium DL-lactate solution (60%, Sigma Aldrich) and 0.5x B-27 Supplement (Gibco). Cells were then harvested using a STEMdiff Cardiomyocyte Dissociation Kit (StemCell Technologies) for downstream experiments.

### 2.2 Gene expression

Samples were collected from independent biological replicates (separate differentiation experiments), and each qPCR assay was run in technical triplicate. Data are presented as the mean of the technical triplicates for each biological replicate. RNA was extracted with the RNeasy Mini kit (Qiagen, Hilden, Germany), and cDNA was synthesized with the High-Capacity cDNA Reverse Transcription Kit (Applied Biosystems, Waltham, MA, United States). Gene expression was measured with the SYBR Select Master Mix (Applied Biosystems) using the ΔCt method, with GAPDH as the housekeeping gene. For normalization, the stage at which the marker is first detected was used as the reference: day 0 for OCT4 expression; day 2 for T (Brachyury); day 4 for KDR; day 8 for GATA4; and day 15 for TNNT2. In addition, day 0 (pluripotent state) served as the reference (control) sample for GPCs. The primers are listed in Supplementary Table S1.

### 2.3 Immunophenotype characterization

Cells were immunophenotyped by flow cytometry on day 15 after differentiation. Cells were fixed and permeabilized using the BD Cytofix/Cytoperm Kit (BD Biosciences, San Diego, CA, United States), following the manufacturer’s instructions. Subsequently, cells were stained with Alexa Fluor 647 mouse anti-cardiac troponin T (BD Biosciences, cat. No. 565,744) for 30 min. Cells stained with Alexa Fluor 647 mouse IgG1 isotype control (BD Biosciences, cat. No. 557,714) were used as negative controls. Samples were analyzed using BD LSRFortessa and FlowJo v10.10.0 software.

The gating strategy included exclusion of debris and non-fixed events, followed by identification of the target cell population using DAPI staining. Cells were further gated based on forward scatter (FSC) and side scatter (SSC) to define size and granularity, and singlets were selected using FSC-A versus FSC-H to exclude doublets. Positivity was defined as fluorescence intensity exceeding that of the corresponding isotype control.

### 2.4 Multichannel electrode array

Extracellular activity of hiPSC-derived cardiomyocytes was recorded using the MEA2100 System (Multichannel Systems, Germany) following established protocols (Mesquita et al., 2023). Cells were plated on fibronectin-coated microelectrode arrays (60MEA200/30iR-Ti; Multichannel Systems) and cultured for 4 days prior to recording, with medium changes every other day. Data acquisition and analysis were performed using Cardio2D V2.14.2 and Cardio2D + V2.9.2 (Multichannel Systems), respectively.

### 2.5 Statistical analysis

Data are shown as mean ± standard deviation. Samples were compared using one-way analysis of variance (ANOVA), followed by Tukey’s multiple comparisons *post hoc* test. A p < 0.05 was considered statistically significant. GraphPad Prism® software version 10 (GraphPad Software Inc., La Jolla, CA, United States) was used for statistical analyses.

## 3 Results and discussion

To explore whether GPCs are dynamically regulated during cardiac lineage commitment, we profiled their temporal expression across key stages of hiPSC cardiac differentiation. A commercially available, chemically defined differentiation kit was used to ensure consistency and reproducibility. Gene expression analysis showed the expected stage-specific and dynamic changes in marker expression throughout differentiation (Figures 1B–F). OCT4 was highly expressed at day 0, consistent with pluripotency, decreased by day 2, and was undetectable by days 4, 8, and 15 (Figure 1B). In contrast, lineage-specific markers emerged sequentially as differentiation progressed. Consistent with this critical early transition, T (Brachyury) expression peaked at day 2 and declined by day 4, marking mesoderm formation and the loss of pluripotency (Figure 1C). KDR expression began on day 4, marking the cardiac mesoderm (Figure 1D), whereas GATA4 expression was initiated on day 8 during cardiac specification (Figure 1E); both markers were maintained through day 15. TNNT2 was first detected at day 8, with robust expression by day 15, consistent with cardiomyocyte differentiation (Figure 1F). Collectively, these findings closely mirror the temporal sequence of gene activation reported in human pluripotent stem cell–based cardiac differentiation systems (Yang et al., 2008; Yilbas et al., 2014; Feeney et al., 2025; Kargaran et al., 2025), supporting the fidelity of our differentiation protocol and confirming appropriate progression through pluripotency exit, mesoderm induction, cardiac specification, and cardiomyocyte differentiation.

Flow cytometry analysis on day 15 demonstrated that 90.83% ± 4.2% of cells expressed cardiac troponin T, confirming successful differentiation (Figure 1G). Spontaneously beating cells were also observed and recorded (Supplementary Video S1). To further characterize the electrophysiological properties of hiPSC-derived cardiomyocytes, multielectrode array (MEA) recordings were performed (Table 1).

**TABLE 1:** Electrophysiological properties of hiPSC-derived cardiomyocytes.

Beating rate (bpm)	Field potential duration (ms)	Amplitude (mV)	Na peak duration (ms)	Na peak max slope (V/s)
21.44 ± 7.60	837.89 ± 277.94	0.70 ± 0.42	8.43 ± 2.49	−0.10 ± 0.08

Bpm, beats per minute, ms, milliseconds, mV, millivolt, V/s, volts per second.

At day 15, cells exhibited an immature electrophysiological phenotype compared with more mature cardiomyocytes (Koivumaki et al., 2018; Carvalho et al., 2022; Weiser-Bitoun et al., 2026), characterized by slower spontaneous beating rates (21.44 ± 7.60 bpm), longer and more variable field potential durations (837.89 ± 277.94 ms), and lower signal amplitudes (0.70 ± 0.42 mV). In addition, cells displayed prolonged sodium peak durations (8.43 ± 2.49 ms) and reduced maximum slopes (−0.10 ± 0.08 V/s) (Table 1), consistent with underdeveloped voltage-gated sodium channel function (n = 201 cells from six biological replicates).

### 3.1 Dynamic expression of glypican isoforms during cardiac differentiation

**FIGURE 2:**
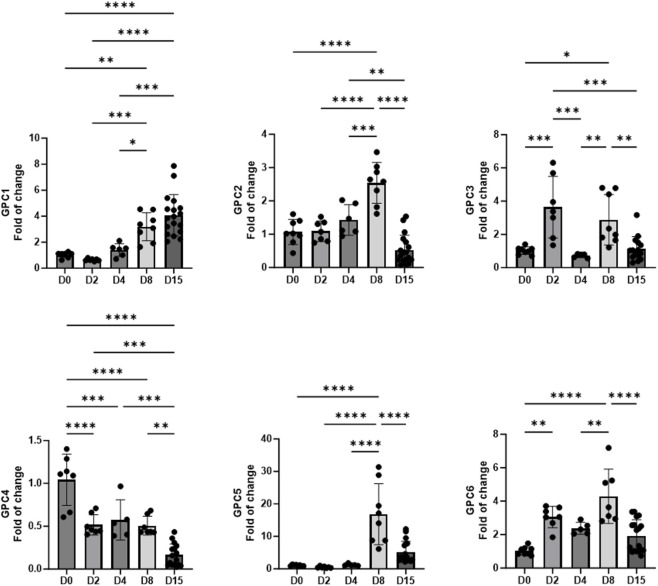
Gene expression of GPCs during cardiac differentiation (n = 6–18). *p < 0.05, **p < 0.01, ***p < 0.001, and ****p < 0.0001.

Analysis of the six human GPC isoforms revealed distinct, stage-associated transcriptional patterns that coincided with specific steps of the differentiation protocol (Figure 2). During the early mesoderm induction phase (day 2), which corresponds to WNT pathway activation, GPC3 and GPC6 were significantly upregulated, whereas GPC4 expression decreased. These reciprocal changes align with developmental studies showing that Gpc4 negatively modulates canonical WNT and BMP signaling during cardiogenesis (Strate et al., 2015). The transient downregulation of GPC4 may thus permit the robust WNT activation required for mesoderm induction. In contrast, the early upregulation of GPC3 and GPC6, both of which are highly expressed in mesodermal embryonic tissues (Veugelers et al., 1999; Shih et al., 2020), suggests that these isoforms may act as coreceptors that facilitate or stabilize WNT and FGF ligand interactions, thereby supporting efficient mesoderm specification.

On day 4, during the WNT-inhibition phase that promotes cardiac progenitor specification, GPC3 expression declined, whereas other isoforms remained relatively stable. This temporal shift mirrors the biphasic role of WNT signaling in cardiac differentiation: initial activation for mesoderm induction, followed by inhibition for cardiac specification (Maas et al., 2023). The dynamic expression of GPC3 and GPC6 during this transition is consistent with a potential role for GPCs in modulating the timing of morphogen activation and repression, which is critical for maintaining cardiac lineage fidelity.

By day 8, when most cells had committed to the cardiac lineage, all GPCs were significantly increased compared with day 0, except for GPC4, which was downregulated. GPC2 and GPC5 showed transient peaks at day 8 and returned to baseline by day 15. Although little is known about the roles of these isoforms in cardiomyocyte biology, they have been implicated in second heart field development, where FGF signaling regulates cardiac progenitor proliferation and specification (Lugert et al., 2017; Takeuchi et al., 2021; Maas et al., 2023). Notably, murine knockdown models of Fgf genes do not exhibit major defects in cardiac specification (Khosravi et al., 2021), suggesting a modulatory rather than essential role. In our study, GPC2 and GPC5 upregulation coincided with cardiac progenitor formation and returned to baseline by day 15, when most cells had differentiated into cardiomyocytes. This expression pattern supports a transient role in early progenitor specification rather than in later cardiomyocyte maturation.

At the final time point analyzed (day 15), as cells matured into cardiomyocytes, GPC1 expression remained elevated, whereas GPC2–6 levels had declined relative to day 8. The sustained expression of GPC1 after lineage commitment points to GPC1’s potential roles in cardiomyocyte homeostasis or electrophysiological regulation. Supporting this hypothesis, Gpc1 knockout mice have cardiac electrophysiological abnormalities associated with altered potassium channel function, such as I_to_ and I_k_ (Souza et al., 2022), as well as reduced systemic blood pressure associated with impaired calcium dynamics (Potje et al., 2021). Thus, GPC1 may contribute to the vascular or electrical regulation of mature cardiac tissue. The sustained expression of GPC1 in hiPSC-derived cardiomyocytes could therefore reflect its involvement in maintaining cellular polarity, membrane organization, or mechano-electrical coupling.

### 3.2 Conserved roles of glypicans in cardiac morphogenesis

The expression dynamics observed in our study align with developmental models that show a conserved role for GPC functions in cardiac morphogenesis. It has been shown that Gpc4 deficiency reduces cardiomyocyte number in zebrafish, and Gpc3 and Gpc6 knockout mice also exhibit congenital heart defects, further supporting a conserved role for GPCs in cardiac morphogenesis (Strate et al., 2015; Filmus, 2023; Mead et al., 2024). The downregulation of GPC4 and the dynamic modulation of GPC3 and GPC6 in our *in vitro* model probably reflect similar regulatory processes during human heart formation.

Interestingly, GPC3 has also been associated with glucose transporter 4 (GLUT4)-mediated glucose uptake (Piao et al., 2025), suggesting potential involvement in metabolic regulation. The sustained GPC3 expression observed at intermediate stages may therefore correspond to the glycolytic metabolic profile characteristic of immature hiPSC-derived cardiomyocytes (Mesquita et al., 2023). As cardiomyocytes mature, they undergo a metabolic shift from glycolysis to fatty acid oxidation, which could underlie the subsequent decline in GPC3 expression by day 15. This observation highlights a potential intersection between GPC signaling and metabolic maturation, an area that remains largely unexplored.

### 3.3 Glypicans as potential temporal modulators of morphogen signaling

**FIGURE 3:**
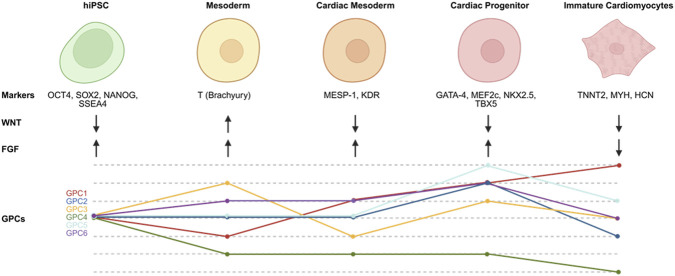
Schematic summary depicting the relationship between GPC expression and morphogen signaling during cardiac differentiation. Major developmental stages, characteristic markers, and temporal dynamics of WNT and FGF pathway activation or inhibition highlight the coordinated regulation of these pathways during differentiation. Created in BioRender. (2025) 412 https://BioRender.com/o8a6hqr.

Collectively, our results demonstrate that glypican family members are dynamically and stage-specifically regulated during human cardiac differentiation, with patterns that coincide with key morphogen-driven transitions. While functional roles were not directly tested, these data provide a descriptive framework that may inform future mechanistic studies examining how extracellular components influence morphogen signaling during cardiac lineage specification. The dynamic and nonredundant expression of GPC1–6 suggests that each isoform plays a distinct role in shaping the signaling microenvironment. Rather than serving merely as structural glycocalyx components, GPCs likely participate in the temporal synchronization of WNT, BMP, and FGF pathway activity, ensuring proper lineage transitions and efficient cardiomyocyte formation (Figure 3). Glypicans’ strategic position on the cell surface allows them to integrate multiple extracellular inputs, effectively functioning as biochemical “modulators” that fine-tune signaling amplitude and duration.

This model has broad implications for the field. By manipulating GPC activity—through targeted overexpression, inhibition, or modification of HS chain sulfation—it may be possible to precisely adjust morphogen signaling to enhance efficiency or maturation. Additionally, understanding the relationship between glycocalyx architecture and morphogen dynamics could lead to more accurate biomimetic culture systems that better replicate the *in vivo* cardiac microenvironment.

## 4 Conclusion

While mechanistic studies are needed to clarify the specific signaling interactions mediated by each GPC isoform, our findings provide the first temporal map of GPC gene expression during human cardiac differentiation. The results establish a foundation for exploring how this family of proteoglycans influences the timing and accuracy of morphogen-driven lineage development. We propose that GPCs constitute an underestimated regulatory layer that links extracellular signaling to intracellular responses, opening new avenues for engineering more physiologically mature hiPSC-derived cardiomyocytes and advancing the field of cardiac regenerative medicine.

## Data Availability

The data supporting the findings of this study are available from the corresponding author upon reasonable request.
