# Supplementary material for: Temporal expression dynamics of glypicans during hiPSC cardiac differentiation
Source: Front Cell Dev Biol. 2026 Feb 25;14:1778977. doi: 10.3389/fcell.2026.1778977 (PMC12975904; doi:10.3389/fcell.2026.1778977)
Supplement: Supplementary file 1 [file Table1.docx]

Supplemental Table 1: List of Primers

| **Primer** | **Forward** | **Reverse** | **Origene #** |
| --- | --- | --- | --- |
| GLYPICAN 1 | GCCCTGACTATTGCCGAAATGTG | GAACTTGTCGGTGATGAGCACC | HP205827 |
| GLYPICAN 2 | GGTTCGTGGCTGTCTCAGCAG | GCAGGTACATCAAACCCTCCGA | HP217936 |
| GLYPICAN 3 | CATTGGAGGCTCTGGTGATGGA | TTGTCCTTCGGAGTTGCCTGCT | HP207790 |
| GLYPICAN 4 | GGAGATGTCGTGAGCAAGGTCT | CATGGCTTCACAGTCACGAGAC | HP205326 |
| GLYPICAN 5 | CGCCAGGATATGCAGCAGTTTC | GCCATGTTCCTGTAGGTACTGC | HP207774 |
| GLYPICAN 6 | TATGGCTCTCCGTGTGATGACC | ACACGTCATCCATGCACCCACT | HP208798 |
| OCT4 | CCTGAAGCAGAAGAGGATCACC | AAAGCGGCAGATGGTCGTTTGG | HP206340 |
| T | CCTTCAGCAAAGTCAAGCTCACC | TGAACTGGGTCTCAGGGAAGCA | HP206752 |
| KDR/VEGFR2 | GGAACCTCACTATCCGCAGAGT | CCAAGTTCGTCTTTTCCTGGGC |  |
| GATA4 | AAGTGTGCGTCTGCCTTTCCCG | TTGTCCGCCTCTGTCTTCTCCA | HP207706 |
| TNNT2 | AAGAGGCAGACTGAGCGGGAAA | AGATGCTCTGCCACAGCTCCTT | HP200344 |
| GAPDH | AATCCCATCACCATCTTCCAG | AAATGAGCCCCAGCCTTC |  |

Supplemental Video 1: Representative recording of spontaneous beating of hiPSC-derived cardiomyocytes. The video was captured using the AmScope 14MP USB 3.0 Color CMOS C-Mount Microscope Camera w Reduction Lens and recorded with AmScope camera software v.10.11.2024.
